# Suppression of NF-κB Activity: A Viral Immune Evasion Mechanism

**DOI:** 10.3390/v10080409

**Published:** 2018-08-04

**Authors:** Liyao Deng, Qiurui Zeng, Mingshu Wang, Anchun Cheng, Renyong Jia, Shun Chen, Dekang Zhu, Mafeng Liu, Qiao Yang, Ying Wu, Xinxin Zhao, Shaqiu Zhang, Yunya Liu, Yanling Yu, Ling Zhang, Xiaoyue Chen

**Affiliations:** 1 Institute of Preventive Veterinary Medicine, Sichuan Agricultural University, Chengdu 611130, China; LiyaoDeng2@163.com (L.D.); mshwang@163.com (M.W.); cqrc_jry@163.com (R.J.); shunchen@sicau.edu.cn (S.C.); zdk24@sicau.edu.cn (D.Z.); liumafengra@163.com (M.L.); yangqiao721521@sina.com (Q.Y.); yingzi_no1@126.com (Y.W.); xxinzhao@sicau.edu.cn (X.Z.); shaqiu86@hotmail.com (S.Z.); yunnyaaliu@163.com (Y.L.); yanling3525@163.com (Y.Y.); zl97451@126.com (L.Z.); 2 Key Laboratory of Animal Disease and Human Health of Sichuan Province, Sichuan Agricultural University, Chengdu 611130, China; chenxy_24@sina.cn; 3 Research Center of Avian Disease, College of Veterinary Medicine, Sichuan Agricultural University, Chengdu 611130, China; 4 School of Medicine, Shanghai Jiao Tong University, Shanghai 200025, China; zengqiurui0128@sjtu.edu.cn

**Keywords:** NF-κB, viruses, NF-κB inhibitors, HIV-1, immune evasion

## Abstract

Nuclear factor-κB (NF-κB) is an important transcription factor that induces the expression of antiviral genes and viral genes. NF-κB activation needs the activation of NF-κB upstream molecules, which include receptors, adaptor proteins, NF-κB (IκB) kinases (IKKs), IκBα, and NF-κB dimer p50/p65. To survive, viruses have evolved the capacity to utilize various strategies that inhibit NF-κB activity, including targeting receptors, adaptor proteins, IKKs, IκBα, and p50/p65. To inhibit NF-κB activation, viruses encode several specific NF-κB inhibitors, including NS3/4, 3C and 3C-like proteases, viral deubiquitinating enzymes (DUBs), phosphodegron-like (PDL) motifs, viral protein phosphatase (PPase)-binding proteins, and small hydrophobic (SH) proteins. Finally, we briefly describe the immune evasion mechanism of human immunodeficiency virus 1 (HIV-1) by inhibiting NF-κB activity in productive and latent infections. This paper reviews a viral mechanism of immune evasion that involves the suppression of NF-κB activation to provide new insights into and references for the control and prevention of viral diseases.

## 1. Introduction

The nuclear factor-κB (NF-κB) family is composed of five related transcription factors: p50, p52, p65, c-Rel, and RelB. The activation of classical NF-κB signaling involves the NF-κB dimer p50/p65, but nonclassical NF-κB signaling involves p52/RelB. In the classical NF-κB signaling pathway, signaling molecules include NF-κB (IκB) kinases (IKKs), IκBα, p50/p65, and IKK upstream receptors and adaptor proteins. The major receptors are toll-like receptors (TLRs), retinoic acid-inducible gene I (RIG-I), tumor necrosis factor (TNF) receptor (TNFR), and interleukin 1 receptor type 1 (IL-1R1), and the major adaptor proteins are myeloid differentiation primary response gene 88 (MyD88), Toll/IL-1 receptor (TIR)-containing adaptor-inducing IFNβ (TRIF), and mitochondrial antiviral signaling protein (MAVS).

The innate immune system is the first line of defense against foreign pathogens. NF-κB plays a major role in innate immune responses by inducing antiviral genes, such as interferon (IFN) and IFN-stimulated genes (ISG). Additionally, NF-κB also promotes viral gene transcription that is harmful to some viruses’ latency. Therefore, over the long course of evolution, viruses have developed multiple methods that interfere with NF-κB activity to promote viral survival.

In this review, we focus on a viral immune evasion mechanism that functions by suppressing NF-κB activation, including targeting receptors, adaptor proteins, IKKs, IκBα, and p50/p65. Additionally, we describe several specific NF-κB inhibitors, including NS3/4, 3C and 3C-like proteases, viral deubiquitinating enzymes (DUBs), phosphodegron-like (PDL) motifs, viral protein phosphatase (PPase)-binding proteins, and small hydrophobic (SH) proteins. To better understand the role of NF-κB in viral immune escape, we use human immunodeficiency virus 1 (HIV-1) as an example, and describe how inhibiting NF-κB activity promotes HIV-1 immune escape in different viral life cycles: productive infection and latent infection. We hope that this review can provide a reference for the prevention and control of viral diseases.

## 2. The Activation of the NF-κB

**Figure 1:**
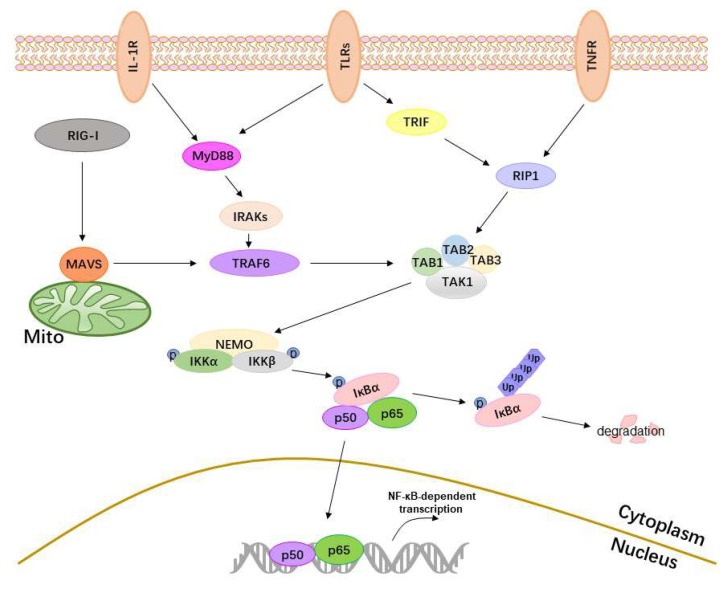
The activation of the NF-κB. The major upstream receptors of NF-κB—TLRs, RIG-I, TNFR, and IL-1R1—sense external stimuli and transmit signals to their adaptor proteins. TLRs transmit signals to MyD88 or TRIF, RIG-I to MAVS, TNFR1 to receptor interacting protein 1 (RIP1), and IL-1R to MyD88. Then, MyD88 activates interleukin-1 receptor-associated kinases (IRAKs) and TNFR-associated factor 6 (TRAF6), MAVS interacts with TRAF6, and TRIF interacts with RIP1. TRAF6 and RIP1 both activate the transforming growth factor (TGF)-β-activated kinase 1 (TAK1) complex. The activated TAK1 complex then activates IKKs, resulting in the phosphorylation and degradation of IκBα and the release of p50/p65. The released p50/p65 enters the nucleus, binds specific DNA sequences, and activates NF-κB transcriptional activity.

Classical activation of NF-κB needs the activation of receptors, adaptor proteins, IKKs, IκBα, and p50/p65 (Figure 1). When cellular receptors sense external stimuli, they transmit signals to the IKKs via adaptor proteins, resulting in the phosphorylation of IKKs, degradation of IκBα, nuclear transfer of p50/p65, and activation of NF-κB.

### 2.1. Receptors

The activation of NF-κB is initiated by receptors, such as TLRs, RIG-I, TNFR, and IL-1R1. All TLRs are type I transmembrane proteins containing three regions: the extracellular, intracytoplasmic, and transmembrane regions. The extracellular region recognizes the external stimulus, and the intracytoplasmic region then transmits signals to downstream adaptor molecules via the TIR domain [1]. TLR3 recruits TRIF, and TLR5, TLR7, and TLR9 recruit MyD88. TLR2 recruits MyD88 and TIR-containing adaptor protein (TIRAP, also known as MAL), and TLR4 recruits MyD88, TIRAP, TRIF, and TRIF-related adaptor molecule (TRAM) [2]. RIG-I contains two N-terminal caspase activation and recruitment domains (CARDs) and a C-terminal domain (CTD) that interacts with the CARDs to prevent unwarranted interactions with downstream factors. However, following binding to nonself RNAs, the interaction between the CTD and CARDs is disrupted, RIG-I undergoes a posttranslational modification by E3 ubiquitin ligase, which promotes K63 polyubiquitination, and RIG-1 reaches an activated state [3,4]. The activated RIG-I then translocates to the mitochondria and mitochondrial-associated membranes, where it interacts with its essential adaptor protein, namely, MAVS. The membrane-bound TNFR1 complex forms within seconds following the engagement of TNFR1 by TNF and independently recruits TNFR-associated death protein (TRADD), TRAFs and RIP1 [5]. IL-1R1 shares some homology with TLRs at regions known as TIR domains, which recruit MyD88 via IL-1 cytokines [6].

### 2.2. Adaptor Proteins

In signal transduction processes, adaptor proteins are critical for activating downstream signals via specific protein–protein interactions, including those involving MyD88, IRAKs, TRIF, MAVS, TRAFs, RIP, and TAK1.

MyD88, the adaptor protein in the TLR/NF-κB pathway, has a C-terminal TIR domain that interacts with the TIR domain of TLRs and an N-terminal death domain (DD), which recruits IRAK4 [7] and then activates IRAK1 and IRAK2. The activated IRAKs dissociate from MyD88 and interact with TRAF6. TRIF acts as an adaptor of TLR3 and TLR4 and contains a TIR domain (residues 380–530), a TRAF6-binding motif (residues 250–255) and a RIP homotypic interaction motif (RHIM) (residues 661–699); the latter two domains interact with TRAF6 and RIP1, respectively [8,9]. MAVS localizes in the mitochondria, peroxisomes, and mitochondrial-associated endoplasmic reticulum membranes (MAMs). This subcellular localization is essential for MAVS signaling function [10,11,12]. MAVS contains an N-terminal CARD-like domain (residues 10–77), an intermediate proline-rich region (PRR) (residues 103–173) and a C-terminal transmembrane (TM) domain (residues 514–535) related to its mitochondrial localization [10]. The N-terminal CARD domain of MAVS interacts with the CARD domain of RIG-I, leading to a conformational change in the MAVS CARD, which, in turn, converts other MAVS into prion-like aggregates [13] and interacts with TRAF2 and TRAF6 via TRAF-interacting motifs [14]. TRAFs are critical adaptors in the NF-κB signaling pathway, and TRAF2, TRAF3, TRAF5, and TRAF6 act as E3 ubiquitin ligases. The activated IRAKs promote the oligomerization of TRAF6 to activate its E3 ubiquitin ligase activity. TRAF6 then autopolyubiquinates, attaching K63 polyubiquitin chains to itself. The K63 polyubiquitin chains of TRAF6 bind to TAK1 kinase complexes, which include TAK1-binding proteins (TABs) and TAK1, and consequently activate TAK1 [15]. RIP1 contains an N-terminal kinase domain (KD), a C-terminal DD and a bridging intermediate domain (ID) containing an RHIM, which is known to mediate homotypic protein–protein interactions. Following the recruitment of E3 ubiquitin ligases, such as TRAF2, to RIP1, RIP1 is ubiquitinated. The K63-linked ubiquitination of RIP1 is recognized by IKK and TAK1 kinase complexes, thus facilitating TAK1-mediated phosphorylation and activation of IKKs [16].

### 2.3. IKKs, IκBα and p50/p65

The IKKs are composed of three major components, namely, IKKα, IKKβ, and NF-κB essential modulator (NEMO). They exist in complex at a ratio of 1:1:2, that is, one IKKα and IKKβ heterodimer binds to a NEMO dimer. NEMO is a ubiquitin-binding protein containing the conserved UBAN (ubiquitin-binding in ABIN and NEMO, where ABIN is the A20-binding inhibitor of NF-κB) domain and preferentially interacts with Met1-linked or Lys63-linked ubiquitin oligomers, which are catalyzed by the linear ubiquitin assembly complex (LUBAC) [17]. IKKα and IKKβ are highly homologous. Both interact with NEMO via the NEMO-binding domain (NBD) and have a kinase domain with two serine residues (S176, S180 for IKKα; and S177, S181 for IKKβ) that require phosphorylation for the kinase to activate NF-κB, a process that is mediated by both TAK1 and IKKβ itself [17,18].

In the resting state, IκBα binds to the Rel homology domain (RHD) of the NF-κB dimer p50/p65 via the ankyrin (Ank) repeat domain, masks the nuclear localization sequence (NLS) of p65, and retains p50/p65 in the cytoplasm. However, activated IKKs phosphorylate IκBα, and phosphorylated IκBα is recognized by the β-transducing repeat-containing protein (β-TrCP), resulting in the Lys48-linked polyubiquitination of IκBα by Skp1-Culin-Roc1-F-box (SCF) E3 ubiquitin ligase complexes (SCF^β-TrCP^) and subsequent degradation.

The degradation of IκBα releases p50/p65. With the help of nuclear transport receptors, which bind to NLS, the free p50/p65 rapidly translocates to the nucleus, where it binds to the κB enhancer and stimulates gene expression. To enhance transcriptional activity, posttranslational modifications of p50/p65, such as phosphorylation, acetylation, methylation, and ubiquitination [19], and coactivators are essential.

## 3. Viruses Suppress NF-κB Activation

During their coevolution with hosts, viruses have acquired effective strategies for suppressing NF-κB activation to escape from immune responses.

### 3.1. Targeting Receptors and Adaptor Proteins

The receptors and their downstream adaptor proteins recognize viruses and transmit signals to NF-κB to induce the host’s antiviral response, and are the first line of defense for the innate immunity. Therefore, a viral immune evasion strategy of NF-κB inhibition is to target the receptors and adaptor proteins. 

The most direct way for viruses to evade immunity is to reduce the production of mRNA and protein levels of receptors and adaptor proteins. Porcine reproductive and respiratory syndrome virus (PRRSV) NSP11 is an endoribonuclease (EndoU) that reduces the mRNA expression levels of both RIG-I and MAVS by its RNase activity, leading to less protein expression of RIG-I and MAVS and subsequently inhibiting NF-κB activation. The three mutants of NSP11 with impaired EndoU activity, H3750A, H3735A, and K3779A, lost the capacity to reduce MAVS RNA expression [20]. Another NF-κB inhibitor, the replication transcription activator protein (RTA) encoded by Kaposi’s sarcoma-associated herpesvirus (KSHV), is an RNA-binding protein that is ubiquitous in γ-herpesviruses and binds to MyD88 RNA, inhibiting the RNA synthesis of MyD88 [21]. However, the nature of the RNA-binding activity and the detailed mechanism for the regulation of MyD88 RNA synthesis require further investigation. Additionally, hepatitis E virus (HEV) open reading frame 3 (ORF3) encodes a protein that blocks the NF-κB signaling pathway through reducing the levels of transcription and translation of TLR4, TRAF6, and nucleotide-binding oligomerization domain containing 2 (NOD2), a receptor associated with the bacterial adaptor molecule [22]. The HEV ORF3 protein also reduces TRADD expression and RIP1 K63-ubiquitination, which is related to the proline-rich domain of ORF3 [23]. Interestingly, MAVS also contains a proline-rich domain that mediates its interactions with TRAFs, RIP1, and Fas-associated death domain protein (FADD), which may be associated with the proline-rich domain of ORF3 mediated NF-κB activation.

A common viral strategy of NF-κB inhibition is to degrade receptors and adaptor proteins via proteasome pathway, blocking the NF-κB signaling pathway, and is employed by KSHV RTA [24,25], the herpes simplex virus-1 (HSV-1) nuclear protein ICP0 [26], the classical swine fever virus (CSFV) NS3 protein [27], the hepatitis B virus (HBV) X protein (HBX) [28], and the coronavirus (CoV) helper protein open reading frame-9b (ORF-9b) [29]. KSHV RTA targets MyD88 and TRIF, HSV ICP0 targets MyD88 and TIRAP, CSFV NS3 targets TRAF6, and HBX and CoV ORF-9b both target MAVS. KSHV RTA is not only an RNA-binding protein but also is identified to be an E3 ligase, and the putative E3 ligase domain is amino acids 118–207. RTA directly interacts with MyD88 to promote polyubiquitination of MyD88, degrading MyD88 via the Ub-proteasome pathway. The RTA mutants C141S and H145L fail to inhibit the accumulation of MyD88 [24]. RTA also degrades TRIF through the proteasome, but that degradation is an indirect interaction, as RTA does not interact with TRIF in co-immunoprecipitation assays [25]. Additionally, RTA reduces TLR2 and TLR4 protein expression, disrupts the membrane localization of TLR2 and TLR4, and strongly inhibits NF-κB activity [30]. RTA may modulate expression of TLR2 and TLR4 via its ubiquitin E3 ligase activity, and it is also possible that RTA modulates expression of TLR2 and TLR4 via transcriptional regulation. It is worth noting that RTA predominantly localizes to the nucleus, whereas MyD88, TRIF, TLR2, and TLR4 are all localized mainly to the plasma membrane and cytosol. Further explanation is needed for how nuclear RTA inhibits cytoplasmic signaling events. Similarly, HSV-1 ICP0 is also a nuclear protein at early times postinfection, and contains a RING domain, which possesses E3 ligase activity to promote the degradation of MyD88 and TRIAP, thus inhibiting the TLR2/NF-κB pathway. The mechanisms of inhibition are likely that (i) newly made ICP0 can directly affect cytoplasmic complexes prior to or as it is being transported to the nucleus; and (ii) small amounts of ICP0 that are present in the cytoplasm are still sufficient to effect the inhibition of TLR2 signaling [26]. 

In addition to reducing the protein levels of receptors and adaptor proteins, viruses also interfere with the functions of receptors and adaptor proteins by interacting with them, thereby inhibiting NF-κB activity. The human cytomegalovirus (HCMV) major tegument protein pUL83 (also known as phosphoprotein pp65) interacts with the interferon-inducible protein IFI16, which acts as a nuclear DNA sensor that is a critical aspect of defense against nuclear replicating viruses, such as herpesviruses. Thus, pUL83 blocks IFI16 pyrin aggregation and subsequent NF-κB signaling [31]. Another DNA sensor, the DNA-dependent activator of the IRFs (DAI), recruits RIP via the RHIM domain. The RHIM-containing murine cytomegalovirus (MCMV) protein M45 interacts with DAI and RIP1 via RHIM–RHIM interaction, blocking NF-κB by disrupting the DAI–RIP1 interaction or inhibiting the ubiquitination of RIP1 [32,33]. M45 also induces the degradation of NEMO by targeting NEMO to autophagosomes for subsequent degradation in the lysosomes [34]. However, hepatitis B e antigen (HBeAg), a nucleocapsid protein of HBV, specifically interacts with the TIR proteins Mal and TRAM, disrupting the homotypic TIR–TIR interaction critical for NF-κB transcriptional activity. The inhibitory effects of HBeAg are dependent on its precore-specific sequence (PSS), which is identified as being similar to the TIR motif [35,36]. HBeAg also inhibits the expression of RIP2, which plays an important role in the activation of NF-κB induced by triggering NOD1, and abolishes NF-κB activity [37]. Furthermore, the human metapneumovirus (hMPV) virulence factor M2-2 not only binds to MAVS to block MAVS–TRAF interaction, but also forms a complex with MyD88, blocking MyD88-induced NF-κB activation [38,39]. M2-2 is enriched with PDZ domains that are common structural domains in signaling proteins for signal transduction and is involved in disrupting MAVS-mediated NF-κB activation.

### 3.2. Targeting IKKs

IKKs contain NEMO, IKKα, and IKKβ and are necessary for IκBα phosphorylation. NEMO acts as a scaffold protein for the IKK complex and mediates interactions with upstream signaling molecules. Therefore, it is effective for viral immune evasion through inhibiting NEMO activity. The modification of NEMO by LUBAC-mediated linear polyubiquitylation is required for the efficient activation of NF-κB. The hepatitis C virus (HCV) nonstructural protein NS3 competes with the binding of NEMO for binding to LUBAC, which results in decreased LUBAC-mediated linear ubiquitylation of NEMO, and thus inhibits the activation of NF-κB [40]. However, the molluscum contagiosum virus (MCV) MC005 protein binds to NEMO in a specific region where NEMO binds IKKα/β, thus inhibiting the activity of the conformational state that NEMO assumes upon binding ubiquitin chains and preventing IKKβ phosphorylation [41]. Additionally, MC159 [42], another MCV protein, the vaccinia virus (VACV) protein C4 [43], and the Orf virus (ORFV)-encoded protein ORFV073 [44] all interact with NEMO to inhibit NF-κB activity. MC159 belongs to a group of proteins collectively known as viral FLICE-like inhibiting proteins (vFLIPs), and was originally defined by the presence of tandem motifs called death effector domains (DEDs), which are used to suppress NF-κB activity by directly interacting with NEMO. However, the mechanisms by which the VACV C4 protein and ORFV073 interact with NEMO need further investigation.

The phosphorylation of IKKα and IKKβ is key for the activation of NF-κB. Therefore, viral proteins that inhibit the phosphorylation of IKKα and IKKβ are indispensable, such as the VACV B14 protein [45,46,47,48], the HCMV tegument protein UL26 [49], the ORFV-encoded protein ORFV024 [50], the MCV MC160 protein [51,52], and the influenza A virus (IAV) NS1 protein [53]. The VACV B14 protein is a Bcl-2-like protein that forms homodimers in the crystal, which is a common feature of the interaction of many viral and cellular Bcl-2-like proteins. B14 interacts with IKKβ via its hydrophobic dimerization interface and sterically hinders the direct contact between the kinase domains of IKKβ, preventing IKKβ phosphorylation and activation [45]. MC160, another MCV vFLIP, is associated with a reduction in IKKα protein levels and the phosphorylation of IKKα and IKKβ, but its DEDs are not required for this function, which is different from MC159-mediated NF-κB activity [51,52]. 

### 3.3. Targeting IκBα

The phosphorylation and subsequent degradation of IκBα are necessary for releasing NF-κB to enter the nucleus. Thus, the porcine epidemic diarrhea virus (PEDV) NSP1 inhibits the phosphorylation and subsequent degradation of IκBα and blocks p65 nuclear translocation. However, NSP1 neither interferes with the phosphorylation of IKKα/IKKβ nor interacts with IκBα or IKKα/IKKβ, suggesting the possible modulation of posttranslational modifications of IκBα, such as SUMOylation, that inhibit the activation of NF-κB [54]. The varicella-zoster virus (VZV) ORF61-encoded protein, a homologous protein of HSV-1 ICP0, prevents β-TrCP-mediated IκBα ubiquitination, which may be associated with the N-terminal RING domain of ORF61 [55]. Finally, there are a class of viral proteins, Ank/F-box proteins, that are likely to be NF-κB inhibitors. For example, the ectromelia virus (ECTV) encodes four Ank/F-box proteins, EVM002, EVM005, EVM154, and EVM165, which all interact with Skp1 and inhibit NF-κB. A possible mechanism underlying this effect is the competition of viral Ank/F-box proteins with β-TrCP for available Skp1 in the SCF^β-TrCP^ complex via the F-box domain. With less available Skp1 in the cell, the interaction between β-TrCP and the SCF^β-TrCP^ complex is diminished [56,57].

### 3.4. Targeting p50/p65 and Reducing NF-κB Transcriptional Activity

The activation of NF-κB requires p50/p65 dimer to enter the nucleus, thus retaining p50/p65 in the cytoplasm as an effective means of inhibiting NF-κB transcriptional activity. Multiple viral proteins bind p50 and p65 to prevent NF-κB from entering the nucleus, including HSV-1 Us3, UL24, UL42, and ICP0 proteins, ORFV-encoded protein ORFV121, enterovirus (EV) 71 2C protein, and MCV MC132 proteins. HSV-1 Us3 protein is a serine/threonine kinase that interacts with p65 and hyperphosphorylates p65 at the site of serine 75 by its kinase activity, thus abolishing p65 nuclear translocation [58]. Us3 also inhibits TLR2-mediated activation of NF-κB through reducing TRAF6 ubiquitination, which could indicate that Us3 recruits cellular or viral deubiquitinases to reverse the ubiquitination of TRAF6 or phosphorylates TRAF6 to prevent the autoubiquitination of TRAF6 [59]. Other proteins encoded by HSV-1, UL24, UL42, and ICP0 all bind to the RHD of p50 and p65 where IκBα binds to p50/p65 [60,61,62], and the EV71 2C protein interacts with the IPI domain of p65, the region dimerized with p50 [63]. In contrast, the domain of p65 interacting with ORFV121 [64] and MC132 [65] is unclear. MC132 interacts with p65 and recruits it to the host Elongin-B/Elongin-C/Cullin-5 ubiquitin ligase complex to cause p65 ubiquitination and subsequent degradation [65]. Interestingly, the murid herpesvirus-4 (MuHV-4) ORF73 protein [66] and its homologous protein, KSHV latency-associated nuclear antigen 1 (LANA-1) [67], both cause p65 ubiquitination and degradation through recruiting p65 to the host Elongin-B/Elongin-C/Cullin-5 ubiquitin ligase complex, but these processes occur in the nucleus, blocking p65 binding to κB sequences.

In the process of activating NF-κB transcriptional activity, the nuclear import of NF-κB is mediated by nuclear transport receptors, such as importin α and importin β. Japanese encephalitis virus (JEV) NS5 protein [68] and Hantaan virus (HTNV) nucleocapsid (N) protein [69] interact with importin α to competitively block the interaction of importin α with its cargo molecule p65, thus inhibiting the nuclear translocation of NF-κB. 

Finally, posttranslational modifications of NF-κB and coactivators are required to enhance NF-κB transcriptional activity. Therefore, reducing the posttranslational modification of NF-κB and blocking coactivator binding are required for successful viral infection. The human bocavirus (HBoV) NS1 inhibits the phosphorylation of p65 at Ser-536, which is induced by IKKβ to promote the binding of p300, a coactivator of NF-κB. Additionally, the NS1 and NS1-70 proteins both interact with nuclear p65 RHD and interrupt the association of NF-κB with its responsive DNA [70]. Another ORFV-encoded protein, ORFV002, physically interacts with p65 and decreases the acetylation of p65 at Lys310, competitively disrupting the interaction between p300 and p65 [71]. CREB-binding protein (CBP) usually interacts with p300, which is a coactivator of NF-κB. The HSV-1 tegument protein VP16 binds to p65, likely sequesters CBP, and then blocks the induction of the NF-κB promoter [72].

## 4. The Specific NF-κB Inhibitors from Viruses

### 4.1. Proteases Encoded by Viruses

Virus-encoded proteases can cleave specific amino acid sites and cut a polypeptide into multiple small peptides. They play key roles in viral replication, such as in the separation of structural and nonstructural proteins, and viral immune evasion by cleaving NF-κB upstream molecules.

#### 4.1.1. NS3/4A

A decade ago, the HCV serine protease NS3/4A was reported to cleave MAVS and TRIF and inhibit the NF-κB signaling pathway and IFN production [73,74]. NS3/4A is a complex composed of NS3 and cofactor NS4A. The consensus cleavage sequence of NS3/4A is D/E-X-X-X-X-C/T|S/A-X-X-X (an acidic aa at the P6 position, a P1 cysteine or threonine, and a P1’ alanine or serine) [75]. The cleavage sequence of TRIF (P-S-S-T-P-C|S-A-H-L) by NS3/4A is similar to the classical cleavage sequence specifically found in NS4B/5A, a viral substrate of NS3/4A, but this TRIF cleavage sequence lacks the acidic P6 residue [74]. However, the MAVS cleavage sequence (E-R-E-V-P-C|H-R-P-S) is not a classical cleavage sequence of NS3/4A. A possible mechanism underlying this effect is that NS3/4A has membrane-targeting domains within NS4A and at the amphipathic helix α_0_ of NS3, is located in intracellular membranes, and targets membrane-anchored proteins. MAVS is a membrane protein, and its C-terminal TM domain determines the correct localization of MAVS [76]. Thus, NS3/4A cleaves MAVS at Cys508 adjacent to the TM domain and dislocates MAVS from the membrane via its membrane-targeting domains [77]. Interestingly, crystal structure analysis shows that the MAVS product complex binding to the NS3/4A active site forms an extensive electrostatic network, which also forms in the NS3/4A viral substrates NS3/4A, NS4A/4B, and NS5A/5B. The electrostatic networks correlate with the affinity of MAVS for binding to NS3/4A and the greater catalytic efficiency of NS3/4A for MAVS vs TRIF and NS4B/5A, which have no such network forms [78]. Additionally, a recent study reports that NS3/4A inhibits the nuclear transport of NF-κB p65 by cleaving importin β1, a nuclear transport receptor. Importin β1 is a novel NS3/4A host substrate that inhibits NF-κB, and the putative cleavage site is at Cys817 [79].

#### 4.1.2. The 3C and 3C-Like Proteases

The picornavirus 3C protease has a typical G-X-C-G motif and a C-H-D/E catalytic center and catalyzes the cleavage of the nonstructural protein of the viral precursor proteins to complete viral replication. At the same time, the 3C protease can cleave important signaling molecules of the NF-κB signaling pathway, including MAVS, TRIF, the TAK1 complex, and NEMO, to inhibit the activation of NF-κB [80,81,82,83,84,85]. The 3C protease has a clear preference for substrate cleavage sites at Q and E, determined by analyzing the cleavage sites of self-polymeric and host proteins [86]. The coxsackievirus (CV) B3 3C protease cleaves MAVS at Q148 and TRIF at Q190, Q653, and Q671 [80]. The EV68 3C protease cleaves TRIF at Q312 and Q653 [81]. EV71 cleaves TRIF at Q312 [87], TAK1 at Q360, TAB2 at Q113, TAB1 at both 414 and Q451, and TAB3 at Q173 and Q343 [82]. The foot-and-mouth disease virus (FMDV) 3C protease cleaves NEMO at Q383 [84], and the hepatitis A virus (HAV) 3C protease cleaves at Q304 [85]. The 3C-like (3CL) proteases NSP4 of PRRSV and NSP5 of PEDV both cleave NEMO, NSP4 at E349 [88], and NSP5 at Q231 [89].

### 4.2. DUBs Encoded by Viruses

Ubiquitination plays an important role in regulating the activation of NF-κB in both a degradation-dependent and a degradation-independent manner. The K48-linked polyubiquitination of IκBα occurs in a degradation-dependent manner to release NF-κB from IκBα. Other types of ubiquitination, including the K63-linked polyubiquitination of RIG-I and TRAF6, K48- and K63-linked polyubiquitination of RIP1, and M1- and K63-linked polyubiquitination of NEMO, occur in a degradation-independent manner to activate signaling molecules [4,90].

#### 4.2.1. Papain-Like Protease (PLP)

The PLPs, which are essential for viral replication, cleave a site within the viral replicase polyproteins and remove ubiquitin from the cellular proteins. The severe acute respiratory syndrome coronavirus (SARS-CoV) PLP removes the Lys63-linked ubiquitin chains of TRAF3 and TRAF6 [91]; the transmissible gastroenteritis virus (TGEV) PLP1 binds and deubiquitinates RIG-I [92]; and PRRSV NSP2, which contains the ovarian tumor (OTU) domain DUB and has been characterized as PLP2, interferes with the polyubiquitination process of IκBα to inhibit the activation of NF-κB [93]. The PLP2 of equine arterivirus (EAV) adopts a compact OTU domain DUB fold with a unique integral zinc finger, which plays a central role in binding and positioning the distal Ub molecule on the protease surface [94]. The zinc-dependent OTU may offer a structure-guided approach for the inhibition of NF-κB by the arterivirus PLP2, but its exact mechanism needs to be further illuminated.

#### 4.2.2. Other Viral DUBs

Multiple herpesviruses encode DUBs, such as HCMV UL48, HSV-1 UL36, KSHV ORF64, and Epstein–Barr virus (EBV) BPLF1, which are all tegument proteins, remove polyubiquitin chains from NF-κB signaling pathway, and inhibit the activation of NF-κB. The HCMV UL48 interacts with RIP1, cleaves its K48- and K63-linked polyubiquitin chains, and inhibits NF-κB activity. During this process, UL45 enhances UL48 activity to cleave RIP1 polyubiquitin chains [95]. The HSV-1 UL36 cleaves polyubiquitin chains from IκBα [96], the KSHV ORF64 reduces the ubiquitination of RIG-I [97], and EBV BPLF1 removes K48-linked ubiquitin chains from IκBα and K63-linked ubiquitin chains from TRAF6 and NEMO [98].

### 4.3. PDL Motifs Encoded by Viruses

The phosphodegron motif DpSGφXpS (GφX refers to a “spacer”, φ stands for a hydrophobic residue, and X is any residue) is recognized by β-TrCP upon phosphorylation of the two serines. IκBα contains the phosphodegron motif DpSGLDpS, which is recognized by the C-terminal WD40 domain of β-TrCP, resulting in the subsequent ubiquitination and degradation of IκBα [99]. Therefore, viruses encode PDL motifs to mimic the IκBα phosphodegron recognized by β-TrCP and interfere with IκBα degradation and the activation of NF-κB.

The rotavirus (RV) NSP1 contains a C-terminal PDL motif (DSGXS) and an N-terminal RING domain. Both serine residues of the PDL motif are phosphorylated by casein kinase II (CKII) in a pattern mimicking phosphorylation of IκBα. This modification is essential for the NSP1 recruitment of β-TrCP. The RING domain interacts with Cullin RING ligase (CRL) complexes, allowing the anchoring of the NSP1-β-TrCP complex to CRL and regulating the ubiquitination of β-TrCP. Thus, NSP1 interferes with IκBα degradation by binding, sequestering or degrading β-TrCP [100,101,102,103]. Amino acids 366 to 372 of the simian varicella virus (SVV) ORF61, a homologous protein of VZV ORF61 and HSV-1 ICP0, represent a PDL motif (LSGPIKS) that is highly similar to DSGφXS. The SVV ORF61 interferes with the ubiquitination of IκBα by binding to β-TrCP, which is likely dependent on the PDL motif [55]. Amino acids 7 to 12 of the VACV protein A49 (SGNLES) and amino acids 52 to 56 of the HIV-1 protein Vpu (SGNES) are both identified as PDL motifs and bind to the WD40 domain of β-TrCP by the PDL motifs, thus diminishing the degradation of IκBα and the activation of NF-κB [104,105,106].

### 4.4. PPase-Binding Proteins Encoded by Viruses

The phosphorylation of IKKs, especially IKKβ, is mediated not only by autophosphorylation and TAK1 but also by PPase [107,108]. Therefore, to suppress NF-κB activity, viruses directly target and dephosphorylate IKKβ by recruiting PPase. The late protein γ_1_34.5 of the HSV-1 and the 2C proteins of EV71, poliovirus (PV), CVA16, and CVB3 all contain PP1-binding motifs that are required for the initial binding of PP1. These PP1-interacting proteins recruit both IKKα/β and PP1, forming a complex to dephosphorylate IKKβ [109,110]. However, the Merkel cell polyomavirus (MCPyV) small T antigen (tAg) recruits a PP4R1/PP4C/PP2A Aβ phosphatase complex, which interacts with NEMO to dephosphorylate IKKs [111,112].

### 4.5. SH Protein Encoded by Viruses

The encoded SH proteins of paramyxoviruses, such as the mumps virus (MuV) [113,114], simian virus 5 (SV5) [113], respiratory syncytial virus (RSV) [115,116], parainfluenza virus 5 (PIV5) [115], J paramyxovirus (JPV) [117], and hMPV [118], have been repeatedly reported to inhibit NF-κB activation. The paramyxoviral SH proteins are type I membrane proteins expressed in the membranes of infected cells and exert similar functions, although they exhibit no sequence homology except for their transmembrane regions. The MuV SH protein coimmunoprecipitates with TNFR1, RIP1, and IRAK1; therefore, the MuV SH protein inhibits the activation of NF-κB by interacting with the TNFR1, IL-1R1, and TLR3 complexes in the membranes of infected cells. Therefore, the mechanism by which paramyxoviral SH proteins inhibit the activation of NF-κB may be related to their membrane localization [114].

## 5. Suppression of NF-κB Activity to Facilitate HIV-1 Immune Evasion

To better understand the viral immune escape mechanism that suppress NF-κB activity in different life cycles, we chose a single virus, HIV-1. AIDS is a highly harmful infectious disease caused by HIV-1, which attacks CD4^+^ T cells and destroys host immune function, seriously threatening human life. Thus, understanding the mechanism of HIV-1 immune evasion by inhibiting NF-κB in productive and latent infections will be beneficial for controlling AIDS. 

In productive infections, HIV-1 infects activated CD4^+^ T cells. Upon entry, HIV-1 goes through reverse transcription, integration, the generation of HIV-1 provirus, virus gene expression, and new virus assembly and budding. However, viral protein expression or newly generated virions are recognized by the host and targeted by the NF-κB-mediated antiviral immune response. Therefore, inhibiting NF-κB activity is required for HIV-1 replication during later stages. Vpu, a late protein of HIV-1, plays a major role in inhibiting the activation of NF-κB in HIV-1 immune evasion. Vpu contains a PDL (serines 52 and 56 of the SGNES sequence), a β-TrCP-binding motif involved in inhibiting NF-κB activation. The mutation of two serines (positions 52 and 56) in HIV-1 NL4.3 Vpu, a lab-adapted HIV-1 strain, completely abolishes its capacity to inhibit NF-κB activity and NF-κB-dependent antiviral gene expression [106,119]. However, the mutation of these serine residues to alanine in primary HIV-1 (CH106) Vpus reduces, but does not fully disrupt, the capacity of the virus to suppress NF-κB [119]. Thus primary HIV-1 Vpu protein shows functional differences from the lab adapted NL4-3 strain, and has other ways to inhibit NF-κB activity other than binding β-TrCP. Tetherin is an interferon-inducible transmembrane protein that has been identified as an inducer of NF-κB activity [120]. Some alanine mutations in the first α-helix of Vpu (i.e., RAE49-51AAA), which is required for efficient Vpu-mediated tetherin counteraction, impair the inhibition of NF-κB activity [119]. This result appears to show that Vpu suppresses NF-κB activity by counteracting tetherin. Therefore, during the viral life cycle, HIV-1 Vpu downmodulates the NF-κB-dependent expression of antiviral proteins at later replication stages to promote viral immune evasion. 

However, if an activated CD4^+^ T cell is infected by HIV-1 during its transition to resting memory T cells, or resting CD4^+^ T cells are directly infected with HIV-1 under endothelial cells simulation, the virus becomes stably integrated into the host cell genome, but cannot produce new virus, which generates HIV-1 latent infection [121]. The integrated provirus is maintained in the latent state by several means, including (a) deleterious mutations in the viral genome; (b) transcriptional interference; (c) changes in chromatin structure; (d) epigenetic silencing; (e) the presence of negative transcription factors; and (f) the absence of positive transcription factors [122]. The positive transcription factors contain NF-κB because the long terminal repeats (LTRs), which act as promoters and enhancers of HIV-1, include κB-binding sequences. When the latently infected resting T cells are exposed to certain cytokines or chemokines, p50/p65 enters the nucleus and binds to HIV-1 LTRs, and the latent virus then transitions into productive infection. Therefore, inhibition the nuclear translocation of p50/p65 is required to establish and maintain HIV-1 latency. 

Cellular HIV-1 Nef-associated factor 1 (Naf1), which inhibits NF-κB activity through ABINs homology domain 2 (AHD2). Naf1 suppresses HIV-1 LTR-driven gene expression by inhibiting the activation of NF-κB because a Naf1 mutant, created by changing two conserved glutamines in AHD2, is unable to inhibit NF-κB and does not suppress HIV-1 LTR-driven gene expression. In contrast, Naf1 knockdown using specific shRNAs significantly increases HIV-1 reactivation upon stimulation with TNFα and phorbol-12-myristate-13-acetate (PMA) in Jurkat T cells [123]. Another negative regulator of HIV-1 replication, identified through whole-genome small interfering RNA (siRNA) screens, is the deubiquitinase cylindromatosis protein (CYLD). In CYLD knockdown cells, NF-κB p65 nuclear translocation significantly increases. In addition, an HIV molecular clone bearing mutations in NF-κB binding sites in the LTR region shows no increase in infection upon CYLD silencing. Therefore, the increased HIV-1 transcription in CYLD knockdown cells is NF-κB dependent [124]. Finally, tripartite motif-containing 32 (TRIM32), an E3 ubiquitin ligase that directly ubiquitinates IκBα and activates NF-κB, promotes reactivation of latent HIV-1 by activating NF-κB [125]. Cellular miR-155 interacts with the 3’-UTR of TRIM32 to suppress its expression, which may promote a return to latency in transiently activated reservoir cells [126]. How HIV-1 suppresses NF-κB activity to establish and maintain latent infection by impacting Naf1, CYLD, or miR-155 needs further investigation, but activating NF-κB activity is an effective “shock and kill” HIV-1 treatment strategy.

**Figure 2:**
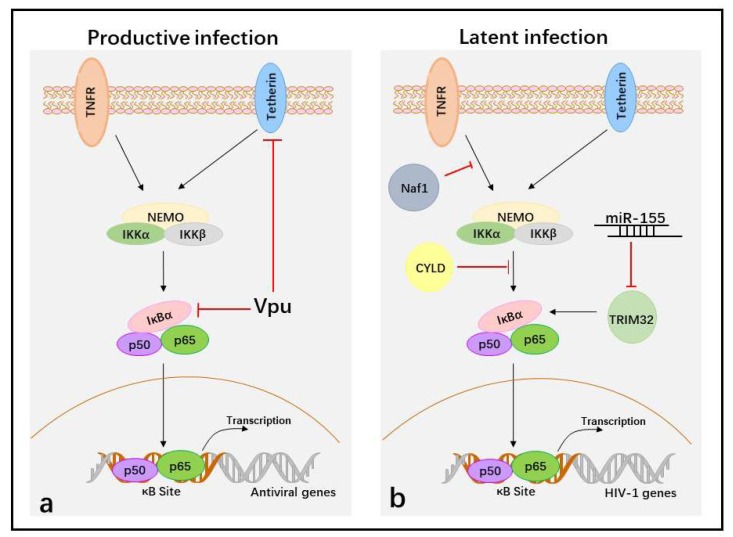
Suppression of the activation of NF-κB to facilitate HIV-1 immune evasion in the different life cycles. (**a**) In productive HIV-1 infection, Vpu protein targets tetherin and IκBα to block NF-κB-dependent antiviral gene transcription during later stages. (**b**) In latent infection, the cellular proteins Naf1 and CYLD inhibit NF-κB activity and block p50/p65 binding at κB sites in HIV-1 LTR to maintain HIV-1 latency, whereas miR-155 blocks p50/p65 binding at κB sites in HIV-1 LTR by targeting TRIM32. The black arrows indicate induction of NF-κB activation and red T bars indicate inhibition of NF-κB activation.

Therefore, suppression of the NF-κB activity is essential for HIV-1 immune evasion in not only productive infection, but also latent infection (Figure 2).

## 6. Conclusions

NF-κB plays a vital role in the antiviral immune response and is a barrier to viral survival. Thus, to survive, it is essential for viruses to suppress NF-κB activity through targeting receptors, adaptor proteins, IKKs, IκBα, and the NF-κB dimer p50/p65 (Table 1). To do so, viruses encode several specific NF-κB inhibitors, including NS3/4, 3C and 3C-like proteases, viral DUBs, PDL motifs, viral PPase-binding proteins, and SH proteins.

It is a common theme that viruses encode multiple proteins to inhibit NF-κB in various ways. For example, HSV-1 encodes multiple NF-κB inhibitors, including ICP0, Us3, UL24, UL36, UL42, VP16, and γ134.5, which impact NF-κB signaling in different ways. ICP0, UL24, and UL42 all interact with p50 and p65, blocking p50/p65 nuclear translocation [60,61,62]. ICP0 also promotes the degradation of MyD88 and TIRAP [26]. In contrast, the tegument protein VP16 binds to p65 in the nucleus, likely sequesters CBP, and then blocks the induction of the NF-κB promoter [72]. Us3, another tegument protein, hyperphosphorylates p65 and reduces TRAF6 polyubiquitination, resulting in suppression of NF-κB [59]. In the end, the late protein γ134.5 recruits both IKKα/β and PP1 to dephosphorylate IKKβ, thus inhibiting the activation of NF-κB [109]. Additionally, HCMV, KSHV, PRRSV, MCV, VACV, and ORFV all encode multiple NF-κB inhibitors to evade immunity. Therefore, viruses inhibit NF-κB activity in multiple ways, which may be a more effective strategy to survive.

However, viral immunopathogenesis is a multistage and complex process that involves a balance between the activation and inhibition of NF-κB. For example, in productive HIV-1 infection, early proteins of HIV-1, Tat [127], and Nef [119], and late protein gp120 [128], all promote HIV-1 replication via the activation of NF-κB. However, the late protein, Vpu, downmodulates NF-κB activity [105,106,119,120]. In fact, it is not difficult to understand that the activation of NF-κB binds to LTRs that facilitate HIV-1 replication early during the viral life cycle, but the host immune system can recognize viral antigens following HIV-1 proliferation. Therefore, to survive, it is necessary to inhibit NF-κB-mediated antiviral immune responses during later stages of infection. Additionally, the inhibition of NF-κB activity is likely a major component of evasion during later stages because Nef and Vpu both express during later HIV-1 stages, when NF-κB activity is inhibited [119]. Therefore, viral immunopathogenesis is a dynamic balance between activation and inhibition NF-κB. 

In this review, we focused on viral immune evasion via the suppression of NF-κB activity, with the goal of providing a reference for the prevention and control of viral diseases.

## Figures and Tables

**Table 1:** Viruses inhibit NF-κB activity.

Virus *	Viral Protein	Mechanisms of Modulation	Host Targets	References
HSV-1	ICP0	Promotes the degradation of MyD88 and TIRAP; binds to RHD of p50 and p65	MyD88, TIRAP, p50, p65	[26,62]
	Us3	Reduces TRAF6 polyubiquitination and hyperphosphorylates p65	TRAF6, p65	[58,59]
	UL24	Binds to the RHD of p50 and p65	p50, p65	[60]
	UL42	Binds to the RHD of p50 and p65	p50, p65	[61]
	VP16	Binds to p65 and probably sequesters CBP	p65	[72]
	UL36	Cleaves polyubiquitin chains from IκBα	IκBα	[96]
	γ134.5	Recruits both IKKα/β and PP1 to dephosphorylate IKKβ	IKKβ	[109]
VZV	ORF61	Prevents β-TrCP-mediated IκBα ubiquitination	IκBα	[55]
SVV	ORF61	Binds to β-TrCP and interferes with IκBα ubiquitination	IκBα	[55]
HCMV	pUL83	Blocks IFI16 pyrin aggregation	IFI16	[31]
	UL26	Decreases phosphorylation of IKKα and IKKβ	IKKα, IKKβ	[49]
	UL48	Cleaves K48- and K63-linked polyubiquitin chains of RIP1	RIP1	[95]
MCMV	M45	Disrupts DAI–RIP1 interactions or inhibits the ubiquitination of RIP1; induces degradation of NEMO	RIP1, NEMO	[32,33,34]
KSHV	RTA	Reduces levels of expressed TLR2, TLR4 and MyD88; degrades MyD88 and TRIF	TLR2, TLR4, MyD88, TRIF	[21,24,25,30]
	LANA-1	Causes p65 ubiquitination and degradation	p65	[67]
	ORF64	Reduces the ubiquitination of RIG-I	RIG-I	[97]
EBV	BPLF1	Removes ubiquitin chains from IκBα, TRAF6 and NEMO	IκBα, TRAF6, NEMO	[98]
MuHV-4	ORF73	Causes p65 ubiquitination and degradation	p65	[66]
PV	2C	Recruits both IKKα/β and PP1 to dephosphorylate IKKβ	IKKβ	[110]
CVA16	2C	Recruits both IKKα/β and PP1 to dephosphorylate IKKβ	IKKβ	[110]
CVB3	2C	Recruits both IKKα/β and PP1 to dephosphorylate IKKβ	IKKβ	[110]
	3C	Cleaves MAVS and TRIF	MAVS, TRIF	[80]
EV71	2C	Interacts with the IPI domain of p65; recruits both IKKα/β and PP1 to dephosphorylate IKKβ	p65, IKKβ	[63,110]
	3C	Cleaves TRIF, TAK1, TAB1, TAB2, and TAB3	TRIF, TAK1, TAB1, TAB2, TAB3	[82,87]
EV68	3C	Cleaves TRIF	TRIF	[81]
FMDV	3C	Cleaves NEMO	NEMO	[84]
HAV	3C	Cleaves NEMO	NEMO	[85]
HBV	HBX	Promotes the degradation of MAVS	MAVS	[28]
	HBeAg	Interacts and colocalizes with Mal and TRAM; inhibits the expression of RIP2	Mal, TRAM, RIP2	[35,36,37]
HCV	NS3	Decreases LUBAC-mediated linear ubiquitylation of NEMO	NEMO	[40]
	NS3/4A	Cleaves MAVS, TRIF, and Importin β1	MAVS, TRIF, Importin β1	[73,74,77,78,79]
HEV	ORF3	Reduces the mRNA levels of TLR4, TLR6, NOD2, and TRADD	TLR4, TLR6, NOD2, TRADD	[22,23]
HBoV	NS1-70	Interacts with p65 RHD	p65	[70]
	NS1	Interacts with p65 RHD and inhibits the phosphorylation of p65	p65	[70]
JEV	NS5	Blocks the interaction of importin α with p65	importin α	[68]
IAV	NS1	Decreases phosphorylation of IKKα and IKKβ	IKKα, IKKβ	[53]
CSFV	NS3	Promotes the degradation of TRAF6	TRAF6	[27]
RV	NSP1	Binds to β-TrCP and interferes with IκBα degradation	IκBα	[100,101,102,103]
PEDV	NSP1	Inhibits the phosphorylation and degradation of IκBα	IκBα	[54]
	NSP5	Cleaves NEMO	NEMO	[89]
PRRSV	NSP11	Reduces the mRNA levels of both MAVS and RIG-I	MAVS, RIG-I	[20]
	NSP4	Cleaves NEMO	NEMO	[88]
	NSP2	Interferes with the polyubiquitination of IκBα	IκBα	[93]
HTNV	N protein	Blocks the interaction of importin α with p65	importin α	[69]
CoV	ORF-9b	Promotes degradation of MAVS	MAVS	[29]
SARS-CoV	PLP	Removes Lys63-linked ubiquitin chains of TRAF3 and TRAF6	TRAF3, TRAF6	[91]
TGEV	PLP1	Binds to and deubiquitinates RIG-I	RIG-I	[92]
hMPV	M2-2	Prevents MAVS from recruiting downstream molecules and interacts with MyD88	MAVS, MyD88	[38,39]
	SH	Unknown	Unknown	[118]
MuV	SH	Interacts with TNFR1, IL-1R1, and TLR3 complexes	TNFR1, RIP1, IRAK1	[113,114]
HIV-1	Vpu	Binds to β-TrCP and diminishes degradation of IκBα, counteracts tetherin	IκBα, tetherin	[105,106,119,120]
MCV	MC005	Inhibits the activity of the conformational state of NEMO	NEMO	[41]
	MC159	Interacts with NEMO	NEMO	[42]
	MC160	Reduces IKKα protein levels and the phosphorylation of IKKα and IKKβ	IKKα, IKKβ	[51,52]
	MC132	Causes p65 ubiquitination and degradation	p65	[65]
VACV	C4	Interacts with NEMO and IKKβ	NEMO, IKKβ	[43]
	B14	Prevents IKKβ phosphorylation and activation	IKKβ	[45,46,47,48]
	A49	Binds to β-TrCP and diminishes degradation of IκBα	IκBα	[104]
ORFV	ORFV073	Inhibits IKK activation, possibly by interacting with NEMO	Unknown	[44]
	ORFV024	Decreases phosphorylation of IKKα and IKKβ	IKKα, IKKβ	[50]
	ORFV121	Binds to p65 and inhibits the phosphorylation of p65	p65	[64]
	ORFV002	Decreases acetylation of p65	p65	[71]
ECTV	EVM002	Interacts with Skp1 via the F-box domain and diminishes the interaction between β-TrCP and the SCFβ-TrCP complex	Skp1	[56,57]
	EVM005			
	EVM154			
	EVM165			
MCPyV	T antigen	Recruits a PP4R1/PP4C/PP2A Aβ phosphatase complex to dephosphorylate IKKs	NEMO	[111,112]

* Abbreviation of each virus is given in the first column of Table 1. HSV-1, herpes simplex virus-1; VZV, varicella-zoster virus; SVV, simian varicella virus; HCMV, human cytomegalovirus; MCMV, murine cytomegalovirus; KSHV, Kaposi’s sarcoma-associated herpesvirus; EBV, Epstein-Barr virus; MuHV-4, murid herpesvirus-4; PV, poliovirus; CAV16, coxsackievirus A16; CVB3, coxsackievirus B3; EV71, enterovirus 71; EV68, enterovirus 68; FMDV, foot-and-mouth disease virus; HAV, hepatitis A virus; HBV, hepatitis B virus; HCV, hepatitis C virus; HEV, hepatitis E virus; HBoV, human bocavirus; JEV, Japanese encephalitis virus; IAV, influenza A virus; CSFV, classical swine fever virus; RV, rotavirus; PEDV, porcine epidemic diarrhea virus; PRRSV, porcine reproductive and respiratory syndrome virus; HTNV, Hantaan virus; CoV, coronavirus; SARS-CoV, severe acute respiratory syndrome coronavirus; TGEV, transmissible gastroenteritis virus; hMPV, human metapneumovirus; MuV, mumps virus; HIV-1, human immunodeficiency virus 1; MCV, molluscum contagiosum virus; VACV, vaccinia virus; ORFV, Orf virus; ECTV, ectromelia virus; MCPyV, Merkel cell polyomavirus.
